# Sensitivity and Specificity of Suspected Case Definition Used during West Africa Ebola Epidemic

**DOI:** 10.3201/eid2401.161678

**Published:** 2018-01

**Authors:** Christopher H. Hsu, Steven W. Champaloux, Sakoba Keïta, Lise Martel, Pepe Bilivogui, Barbara Knust, Andrea M. McCollum

**Affiliations:** Centers for Disease Control and Prevention, Atlanta, Georgia, USA (C.H. Hsu, S.W. Champaloux, L. Martel, B. Knust, A.M. McCollum);; Prevention and Disease Control, Conakry, Guinea (S. Keïta);; Surveillance of National Ebola Coordination Cell, Conakry (P. Bilivogui)

**Keywords:** Ebola virus disease, Ebola viruses, viruses, sensitivity, specificity, suspected case definition, epidemic, zoonoses, West Africa, Guinea

## Abstract

Rapid early detection and control of Ebola virus disease (EVD) is contingent on accurate case definitions. Using an epidemic surveillance dataset from Guinea, we analyzed an EVD case definition developed by the World Health Organization (WHO) and used in Guinea. We used the surveillance dataset (March–October 2014; n = 2,847 persons) to identify patients who satisfied or did not satisfy case definition criteria. Laboratory confirmation determined cases from noncases, and we calculated sensitivity, specificity and predictive values. The sensitivity of the defintion was 68.9%, and the specificity of the definition was 49.6%. The presence of epidemiologic risk factors (i.e., recent contact with a known or suspected EVD case-patient) had the highest sensitivity (74.7%), and unexplained deaths had the highest specificity (92.8%). Results for case definition analyses were statistically significant (p<0.05 by χ^2^ test). Multiple components of the EVD case definition used in Guinea contributed to improved overall sensitivity and specificity.

The 2014–2016 West Africa Ebola virus disease (EVD) epidemic became the largest filovirus outbreak in history; 28,646 reported cases (suspected, probable, and confirmed) and 11,323 deaths were reported as of March 30, 2016 (*1*). Early identification of suspected EVD cases was needed to prevent additional persons from becoming infected and stop the epidemic. However, a major difficulty in correctly identifying cases is that nonspecific symptoms associated with EVD mimic those of other common febrile diseases, such as malaria and typhoid fever. The early course of EVD might include general signs and symptoms, such as headache, fever, chills, and myalgia, which can progress to vomiting, diarrhea, hemorrhage, shock, and end organ failure (*2*,*3*). Furthermore, variations in clinical presentations between persons can complicate case identification (*4*). Fever, vomiting, diarrhea, and hemorrhage are the most familiar signs associated with EVD. However, fever was either not reported or not observed in 11%–24% of reported cases, and the presence of hemorrhagic symptoms varied widely (1%–51%) (*4*–*10*). Nonetheless, early identification and aggressive supportive care early in an infection might improve the outcome of patients (*4*).

Development and use of appropriate case definitions can help identify suspected EVD cases early. This identification can in turn reduce the number of persons exposed to an infectious patient and ensure quality supportive care early in the illness of a patient. Furthermore, a proper case definition is not only needed from an epidemiologic classification standpoint but also has downstream implications related to identifying cases, controlling an outbreak, and saving lives.

A case definition with a high type 1 error rate (false-positive results) could potentially result in unnecessary exposure of misclassified patients in an Ebola treatment unit (ETU). Likewise, a case definition with a high type 2 error rate (false-negative results) can result in further exposures and infections (e.g., an infected but undetected patient in a community). For these reasons, different case definitions have been developed for EVD, depending on the *Ebolavirus* species and the goals of surveillance.

As an illustration, 1 approach is a highly sensitive (i.e., broad) clinical case definition that enables all possible signs and symptoms of EVD to be detected, with confirmation relying on highly specific diagnostic testing. This approach can be valuable in a setting in which diagnostic testing and healthcare facilities are available to test and safely care for all persons who satisfy the case definition. Another approach is the use of a more stringent clinical case definition for EVD for patients who do not have known risk factors (i.e., contact with EVD cases) and enables a lower threshold for suspecting EVD if a person has had risk for exposure. This strategy could be essential in resource-limited areas where testing facilities are not readily available or where there might be delays in laboratory results. Rapid detection of EVD and institution of appropriate infection control procedures in these areas rely heavily on quick patient identification and presumptive diagnosis before laboratory confirmation.

Multiple case definitions were used during the West Africa EVD epidemic, as exemplified by EVD surveillance in Sierra Leone, Liberia, and Guinea, which each used variations of the suspected case definition (*9*,*11*–*14*). A commonly used suspected case definition used in Guinea was developed by the World Health Organization (WHO) (*15*). We describe the diagnostic performance of this suspected case definition by using epidemiologic surveillance and diagnostic test data for Guinea.

## Methods

### Case Definition

The WHO suspected case definition was used in Guinea, and similar versions were used throughout West Africa during the epidemic. This definition was defined as 1) any person, alive or dead, who has (or had) sudden onset of high fever and contact with a suspected, probable, or confirmed EVD case-patient, or a dead or sick animal; or 2) any person with sudden onset of high fever and >3 signs/symptoms (headache, generalized or articular pain, intense fatigue, nausea/vomiting, loss of appetite, diarrhea, abdominal pain, difficulty swallowing, difficulty breathing, hiccups, miscarriage); 3) unexplained bleeding; or 4) sudden unexplained death.

### Population Dataset

As part of national surveillance for EVD in West Africa, a standard case investigation form was completed for all patients who were suspected of having EVD (*16*), and diagnostic laboratory testing was conducted for patient specimens. Confirmed case-patients were persons who had positive results for Ebola virus (EBOV) RNA by reverse transcription PCR. Non–case-patients were persons tested for EVD and who had negative results by reverse transcription PCR (*17*). These data were compiled nationally by the Guinea Ministry of Health by using the viral hemorrhagic fever application in Epi Info version 7.1.4 (Centers for Disease Control and Prevention, Atlanta, GA, USA), which was put into operation in Guinea in April 2014 (*18*).

For this analysis, we used deidentified national line list data for all persons with symptom onset or case detection dates during March−October 2014, which was during the early stage of the EVD outbreak. Analyses were limited to this period because all the analyses in this study were conducted in 2015, during the EVD response. From a database of 3,216 persons, we excluded 369 (11.5%) who were missing complete case report forms or laboratory reports. This exclusion resulted in a dataset of 2,847 persons for further investigation.

Initial clinical signs/symptoms and associated epidemiologic risk factors (contact with infected persons or body fluids, handling of bushmeat, attending the funeral of an Ebola case-patient) were presented mostly in closed response formats and had yes, no, and unknown response categories. The clinical data used in the study were generally captured at the date of initial case identification. We conducted 2 types of analyses by using the 2,847 persons who had complete case report forms and laboratory reports: 1) a complete case definition analysis that required satisfying >1 of the 4 criteria (epidemiologic risk factor criteria, clinical criteria, unexplained bleeding criteria, and unexplained death criteria); and 2) individual criteria analyses where each of the 4 criteria was assessed separately. For individual criteria analyses, if a person had missing data in the specific criteria of interest, we did not include them in the analysis. Deaths were surmised as unexplained death if the person was declared dead at the time the case report form was completed but no cause of death was reported (n = 157). Data cleaning ensured proper French to English language conversion and that all components were linked to the appropriate patient, including epidemiologic risk factors, laboratory samples, and laboratory test results.

### Statistical Analysis

We used SAS software (SAS Institute Inc., Cary, NC, USA) to conduct complete case definition analysis and individual criteria analyses. We calculated sensitivity, specificity, positive predictive value (PPV), and negative predictive values (NPV). We used a χ^2^ test to determine whether the case definition and laboratory confirmation were significantly associated (p<0.05).

## Results

Of the 2,847 persons included in the analysis, 14.9% were <15 years of age and 14.5% were >55 years of age. Within this dataset, 53.5% of persons were reported to have had a fever (tactile or measured); however, only 63 (2.2%) persons had specific temperature readings recorded (Table 1). Fever, fatigue, diarrhea, and nausea/vomiting were the most commonly reported signs/symptoms. Among persons with recorded final outcome data (n = 2,136, 75%), 52.6% died. A total of 1,304 case-patients (45.8%) had a record indicating that laboratory tests confirmed EBOV infection, and 17.3% of participants reported >1 epidemiologic risk factor.

**Table 1:** Characteristics of 2,847 persons used for analysis of suspected case definition during West Africa Ebola epidemic, Guinea, March–October 2014

Characteristic	No. (%)
No. persons	2,847 (100.0)
Sex	
M	1,396 (49.0)
F	1,408 (49.5)
Missing	43 (1.5)
Age, y	
0–<5	166 (5.8)
5–15	259 (9.1)
16–25	474 (16.6)
26–35	625 (21.9)
36–45	517 (18.2)
46–55	295 (10.4)
>55	414 (14.5)
Missing	97 (3.4)
Fever (temperature) recorded	
>38.6°C	29 (1.0)
>38°C	34 (1.2)
Missing	2,813 (98.8)
Sign/symptom reported	
Fever, tactile or measured at >38°C	1,524 (53.5)
Fatigue	1,400 (49.2)
Diarrhea	963 (33.8)
Vomiting/nausea	1,019 (35.8)
Anorexia	772 (27.1)
Headache	782 (27.5)
Chest pain	44 (1.5)
Abdominal pain	483 (16.9)
Muscle pain	445 (15.6)
Joint pain	331 (11.6)
Difficulty swallowing	32 (1.1)
Difficulty breathing	35 (1.2)
Hiccups	121 (4.3)
Unexplained bleeding	274 (9.6)
Epidemiologic risk factors in past month	
Contact with any sick person	282 (9.9)
Contact with infectious bodily fluids from known case-patient	36 (1.3)
Physical contact with a case-patient, dead or alive	87 (3.1)
Participated in a funeral touching/carrying a body	83 (2.9)
Contact with bats or nonhuman primates	2 (0.1)
Laboratory confirmation status	
Reverse transcription PCR positive	1,304 (45.8)
Reverse transcription PCR negative	1,233 (43.3)
Unconfirmed/eliminated	310 (10.9)
Final status	
Alive	1,012 (35.5)
Dead	1,124 (39.5)
Status missing	711 (24.9)

### Complete Case Definition Performance

Approximately half of the persons in the dataset (1,412 [49.6%]) had complete data fields to satisfy >1 of the 4 field case definition criteria (epidemiologic, clinical, unexplained bleeding, or unexplained death) to be included in the analysis of the complete case definition. A total of 801 persons had confirmed cases; 611 persons were classified as having noncases. A total of 552 (64.2%) cases and 308 (35.8%) noncases satisfied the complete definition (Table 2). The sensitivity was 68.9%, the specificity was 49.6%, the PPV was 64.2%, and the NPV was 54.9% (p<0.0001) (Table 3).

**Table 2:** Analysis of complete Ebola virus disease suspected case definition and individual criteria during West Africa Ebola epidemic, Guinea, March–October 2014*

Criteria choice	Cases, no. (%)	Noncases, no. (%)
Meets complete definition	552 (64.2)	308 (35.8)
Does not meet complete definition	249 (45.1)	303 (54.9)
Meets epidemiologic risk criteria†	121 (82.3)	26 (17.7)
Does not meet epidemiologic risk criteria	41 (43.6)	53 (56.4)
Meets clinical criteria	458 (66.4)	232 (33.6)
Does not meet clinical criteria	343 (47.5)	379 (52.5)
Meets unexplained bleeding criteria	79 (49.1)	82 (50.9)
Does not meet unexplained bleeding criteria	722 (57.7)	529 (42.3)
Meets unexplained death criteria	113 (72.0)	44 (28.0)
Does not meet unexplained death criteria	683 (54.8)	564 (45.2)
*Percentages correlate with rows. †Contact with infected persons or body fluid, handling of bushmeat, attending the funeral of an Ebola case-patient.		

**Table 3:** Analysis of Ebola virus disease suspected case definition during West Africa Ebola epidemic, Guinea, March–October 2014

Component of case definition analyzed	p value	Sensitivity, %	Specificity, %	Positive predictive value, %	Negative predictive value, %
Complete definition	<0.0001	68.9	49.6	64.2	54.9
Epidemiologic risk criteria*	<0.0001	74.7	67.1	82.3	56.4
Clinical criteria	<0.0001	57.2	62.0	66.4	52.5
Unexplained bleeding	0.04	9.9	86.6	49.1	42.3
Unexplained death	<0.0001	14.2	92.8	72.0	45.2

*Contact with infected persons or body fluid, handling of bushmeat, attending the funeral of an Ebola case-patient.

### Epidemiologic Criteria Performance

For the epidemiologic risk factor criteria, 241 (8.5%) of 2,847 persons had complete data fields for the analysis. A total of 162 persons had confirmed cases; 79 persons were classified as having noncases. A total of 128 (82.3%) cases and 26 (17.7%) noncases satisfied the epidemiologic risk factor criteria (Table 2). The sensitivity was 74.7%, the specificity was 67.1%, the PPV was 82.3%, and the NPV was 56.4% (p<0.0001) (Table 3).

### Clinical Criteria Performance

For the clinical criteria component, 1,412 (49.6%) of 2,847 persons had complete data fields for the analysis. A total of 801 persons had confirmed cases; 611 persons were classified as having noncases. A total of 458 (66.4%) cases and 232 (33.6%) noncases satisfied the clinical criteria (Table 2). The sensitivity was 57.2%, the specificity was 62.0%, the PPV was 66.4%, and the NPV was 52.5% (p<0.0001) (Table 3).

### Unexplained Bleeding Criteria Performance

For the unexplained bleeding criteria, 1,412 (49.6%) of 2,847 persons had complete data fields for the analysis. A total of 801 persons had confirmed cases; 611 persons were classified as having noncases. A total of 79 (49.1%) cases and 82 (50.9%) noncases satisfied the unexplained bleeding criteria (Table 2). The sensitivity was 9.9%, the specificity was 86.6%, the PPV was 49.1%, and the NPV was 42.3% (p = 0.04) (Table 3).

### Unexplained Death Criteria Performance

For the unexplained death criteria, 1,404 (49.3%) of 2,847 persons had complete data fields for the analysis. A total of 796 persons had confirmed cases; 608 persons were classified as having noncases. A total of 113 (72%) cases and 44 (28%) noncases satisfied the unexplained death criteria (Table 2). The sensitivity was 14.2%, the specificity was 92.8%, the PPV was 72.0%, and the NPV was 45.2% (p<0.0001) (Table 3).

## Discussion

This analysis examined the performance of a case definition used for surveillance during the West Africa EVD epidemic. Developing appropriate case definitions in the setting of an outbreak or epidemic is critical because of the need to balance the strengths of the definition (good sensitivity, specificity, PPV, and NPV) with the utility of the definition in the particular setting. A previous analysis of a simplified 1999 WHO case definition (which consisted of fever and unexplained hemorrhage as a suspected case definition) found that these simple criteria resulted in poor sensitivity and a misclassification of 30% of cases infected with Ebola virus or Marburg virus (*14*). In contrast, our analysis of a complex multipart case definition found a relatively higher sensitivity (68.9%). A more recent study analyzed the diagnostic validity of EVD clinical features of the WHO suspected case definition for patients admitted to an ETU during the second half of the epidemic in Sierra Leone (*19*). That study found that the epidemiologic risk factors (previous contact with an EVD case-patient) were strongly correlated with EVD diagnosis and that the suspected case definition showed low specificity and PPV, in agreement with our analysis.

The complete case definition showed poor sensitivity (68.9%) and specificity (49.6%). However, the case definition included subcriteria that showed higher sensitivity and specificity when analyzed individually. Among the 4 criteria, unexplained death (92.8%) and unexplained bleeding (86.6%) had the highest specificity, which indicated that if these patients were identified and tested in the EVD outbreak setting, there was higher likelihood of being EVD laboratory confirmed. The lower sensitivity in the complete case definition could have been affected by the epidemiologic risk criteria for which, among the 2,847 persons evaluated, only 241 (8.5%) had epidemiologic information for assessing whether they satisfied the epidemiologic risk criteria. The incomplete, missing data might have caused a bias. If the dataset had a high proportion of persons who had complete epidemiologic risk criteria, then the complete case definition might have a higher sensitivity and specificity than the subcriteria.

The reasons for a death being classified as unexplained could be caused by incomplete or erroneous data entry of case report forms or poor recall of patient history. It is essential to note that information about unexplained deaths was not reported in the outbreak case report forms. Rather, this criterion was artificially created on the basis of absence of clinical or epidemiologic data, or a cause of death provided, so that we could approximately capture this information for our analysis. The high number of unexplained deaths could reflect the high number of overall deaths reported during the early portion of the EVD epidemic, in which surveillance had potential lags, leading to patients not being detected until they had died.

Although the unexplained bleeding criteria showed a relatively high specificity (86.6%), the sensitivity was the lowest among the analyses (9.9%). These findings could be explained by the observation that although bleeding is certainly a striking presentation for EVD patients, medical documentation in which it was reported that the patient had bleeding varied from 2% to 69%. Lower percentages were reported in nonobservable hemorrhage (e.g., gastrointestinal bleeding) and for survivors (*3*,*4*,*10*). These findings could also explain the low PPV (49.1%) and NPV (42.3%) of unexplained bleeding criteria in this analysis.

The presence of epidemiologic risk factors had the highest sensitivity (74.7%) and high PPV (82.3%), indicating that most laboratory-confirmed case-patients had a reported exposure to EVD case-patients, which is consistent with previous investigations of EBOV transmission (*20*–*22*). However, this subcriteria also had the smallest sample size when compared with other subcriteria. Response bias, as a result of fewer cases with available epidemiologic data, could have caused epidemiologic risk factors to perform better than the other subcriteria. We surmise that if the sample size for epidemiologic risk factors was similar to those for other subcriteria, its performance would not be as high. Along these lines, accurate assessments of exposure of a person to EBOV is not always possible, and it is necessary to include clinical criteria as a component of a case definition. The performance of the clinical criteria was moderate, as might be expected because EVD is known to be difficult to diagnose by only clinical criteria, but performance is especially needed when epidemiologic information might be lacking (*9*). The clinical criteria often guide clinicians to order tests for laboratory confirmation of EVD. This procedure also reduces the risk for missing possible EVD patients and discharging them from treatment where they could shed virus in the community.

The case definition analyzed in this study was implemented in the midst of an unprecedented outbreak. A major advantage of this complete case definition was its broad and complex scope that enabled wider inclusion of suspected cases. The definition enabled persons to be considered as having suspected cases if they did not meet clinical criteria but had an epidemiologic link with another case-patient or if there was an unexplained death. Broad inclusive criteria are needed to identify cases and immediately control further spread of the virus. Once a suspected case is identified, resource-intensive contact tracing can begin even while laboratory confirmation is pending.

The benefits of the Guinea dataset used in our analyses were the large sample size and level of detail. These features enabled an in-depth analysis to determine if specific components of the case definition played roles in their performance. However, the dataset also had limitations. First, it is unknown how representative this database was of all EVD patients. This dataset was dependent on patients identified early in the Ebola outbreak when case identification was still being established. Developing the suspected case definition during the early stages of the outbreak could have been dependent on what commonalities were observed among infected patients, which could also mean some patients who did not exhibit the common presentation could be missed. Therefore, this limitation could cause an overestimation of the performance characteristics in our analyses.

In addition, because the dataset was collected early in the response, the quality of the data collected could be suspect. Many portions of the data were missing, and clinical symptoms and epidemiologic information could have been inaccurate. For example, few patients reported epidemiologic risk factors that could be a source of misclassification bias, which would lead to inaccurate measures of sensitivity and specificity. Also, clinical and epidemiologic data might not have been completely or consistently collected because of various levels of training and a large number of personnel who completed the case report forms. Despite these challenges, future EVD outbreaks would benefit in training public health staff in thorough, consistent data collection and documentation to reduce this problem. Finally, this analysis assessed only 1 case definition used in West Africa. Other case definitions used during the epidemic will perform differently on the basis of the criteria.

Although individual criteria had their strengths in sensitivity and specificity compared with the complete case definition, none should be mutually exclusive. The need for detecting every suspected case in a large EVD epidemic was reliant on a broad case definition that included criteria from all possible scenarios. A complete case definition was especially appreciated when epidemiologic and medical histories for patients were often incomplete or lacking during the height of the epidemic; several cases that were laboratory confirmed did not meet criteria for the complete case definition. An explanation for this dichotomy would be that in the midst of an epidemic, when the threshold to diagnose a possible EVD case was low, clinicians were often prompted to test a patient for EVD even if the case definition was not satisfied. The concern for missing a suspected case might have outweighed strict efforts to follow the case definition. Furthermore, the poor specificity of the case definition reflects its development for public health and epidemiologic purposes and not for clinical screening in the setting of triage (*14*,*23*,*24*). However, because identifying a suspected case could lead the patient to a clinical setting, such as a suspected case-patient arriving at an ETU, its utility should not be limited to only epidemiology. Thus, use of a case definition should not be rigid but should be used as an adaptable tool.

The use of the case definition is a cogent starting point to identify possible cases and, as more information is gathered (i.e., signs, symptoms, clinical status), can eventually aid clinical management decisions. Similarly, case definitions should not be the end-all deciding factor on clinical management of a patient. For example, decisions on admission and treatment for critically ill patients with an illness that might meet the suspected case definition but that is clearly not EVD, such as measles, who arrive at an ETU should be balanced with the clinical judgment of the physician, risk/benefit to patients, and available resources.

Likewise, the sensitivity and specificity of a case definition are not only affected by the criteria included in the definition but by its use during the timing of an outbreak. In the early stages of an outbreak, when case reporting might still be established, it might be more useful to identify true cases. However, in the final stages of the outbreak and as the response is better established, an increase in sensitivity to identify all possible cases might offset a loss of specificity. Therefore, understanding these dynamics is needed in the deployment of appropriate case definitions.

In Guinea, the case definition for suspected cases was last revised in April 2016 to take into consideration the lessons of the last outbreak in Koropara and related new scientific knowledge highlighting the possibility of sexual transmission from the sperm of an Ebola survivor >1 year after his release from an ETU (*25*). Therefore, since that time, the new definition has been broadened to include 2 additional individual epidemiologic conditions: 1) 2 deaths in 1 family in a period of 3 weeks, or 2) 1 death in the family or acquaintances of an Ebola survivor. Also, because fever measurement was missing for 99% of the persons in the dataset, a future consideration is how fever and other clinical criteria (e.g., headache, generalized or articular pain, nausea/vomiting) might be useful factors in the definition of a suspected case of EVD. This exploration should be a fruitful endeavor, especially since the presence of fever is assumed to be an integral part of EVD case definitions.

Modification of surveillance databases to include new variables that take into account evolving case definitions and completeness of data will remain a challenge worth pursuing. Frequent diagnostic performance evaluation and revision of case definitions by using available data have the potential to play a major role in early identification of cases and related improved outcomes. Therefore, understanding of these dynamics is needed during all stages of an outbreak.
